# Antiretroviral Therapy in the Malawi Defence Force: Access, Treatment Outcomes and Impact on Mortality

**DOI:** 10.1371/journal.pone.0001445

**Published:** 2008-01-16

**Authors:** Alfred C. Banda, Simon D. Makombe, Andreas Jahn, Hannock Tweya, Stuart Chuka, Joseph Kwong-Leung Yu, Bethany Hedt, Ralf Weigel, Amon Nkhata, Erik J. Schouten, Kelita Kamoto, Anthony D. Harries

**Affiliations:** 1 Malawi Defence Force, Medical Department, Lilongwe, Malawi; 2 HIV Unit, Ministry of Health, Lilongwe, Malawi; 3 Lighthouse Trust, Lilongwe, Malawi; 4 International Training and Education Center on HIV, Seattle, Washington, United States of America; 5 Malawi Business Coalition against AIDS, Blantyre, Malawi; 6 Taiwan Medical Mission, Mzuzu Central Hospital, Mzuzu, Malawi; 7 Harvard School of Public Health, Boston, Massachusetts, United States of America; 8 Management Sciences for Health, Lilongwe, Malawi; 9 Family Health International, Malawi Country Office, Lilongwe, Malawi; 10 London School of Hygiene and Tropical Medicine, London, United Kingdom; McGill University AIDS Centre, Canada

## Abstract

**Background:**

HIV/AIDS affects all sectors of the population and the defence forces are not exempt. A national survey was conducted in all public and private sectors in Malawi that provide antiretroviral therapy (ART) to determine the uptake of ART by army personnel, their outcomes while on treatment, and the impact of ART on mortality in the Malawi Defence Force.

**Methodology/Principal Findings:**

A retrospective cohort analysis was carried out, collecting data on access and retention on treatment from all 103 public and 38 private sector ART clinics in Malawi, using standardised patient master cards and clinic registers. Observations were censored on December 31^st^ 2006. Independent data on mortality trends in army personnel from all causes between 2002 and 2006 were available from army records. By December 31^st^ 2006, there were 85,168 patients ever started on ART in both public and private sectors, of whom 547 (0.7%) were army personnel. Of these, 22% started ART in WHO clinical stage 1 or 2 with a CD4-lymphocyte count of ≤250/mm^3^ and 78% started in stage 3 or 4. Treatment outcomes of army personnel by December 31^st^ 2006 were:−365 (67%) alive and on ART at their registration facility, 98 (18%) transferred out to another facility, 71 (13%) dead, 9 (2%) lost to follow-up, and 4 (<1%) stopped treatment. The probability of being alive on ART at 6-, 12- and 18-months was 89.8%, 83.4% and 78.8% respectively. All-cause mortality in army personnel declined dramatically over the five year period from 2002–2006.

**Conclusion/Significance:**

There has been a good access of army personnel to ART during the last five years with excellent outcomes, and this should serve as an example for other defence forces and large companies in the region.

## Introduction

Malawi has been scaling up antiretroviral therapy (ART) since 2004, and good progress has been made [1]. Treatment is available free of charge in the public sector, and can also be obtained at a subsidised rate from the private sector. Data on all ART patients in both public and private sectors are recorded using a standardised system for monitoring case finding and treatment outcomes [2], and this includes information on occupation. The system is rigorously supervised every three months [3], and the data can be considered nationally complete and reliable, offering the opportunity to examine uptake, retention in therapy and survival for subgroups of the population.

The Malawi Defence Force has been affected by the HIV epidemic in the same way as the rest of the population. Before national scale up of ART started, some infected army personnel were offered ART, the drugs paid for from the Defence Force Budget. When ART scale up started in 2004, three army medical clinics were included in the first round of sites, and a fourth army clinic was added in mid-2005. Army personnel, their families and the surrounding catchment population can receive free ART from any of these four clinics as well as from any public or private sector site in the country.

Given the importance of maintaining healthy personnel in the country's defence force, we were interested to know how many army personnel had accessed ART, their outcomes while on treatment, and whether ART had had any impact on mortality rates in general in the Defence Force.

## Methods

### Background

From June 2004 onwards, ART was made free within the public sector and national roll-out started. The details have been previously reported [2], and are only briefly described. A simple, standardised approach is used which focuses on:- one generic, fixed dose combination treatment (stavudine, lamivudine, nevirapine); a standardised system of registration, monitoring and reporting of cases and outcomes; and quarterly monitoring and supervision visits to all ART sites. Adult patients are eligible for ART if they have a positive HIV test, understand the implications of therapy, and are assessed in WHO clinical stage 3 or 4 or have a CD4-lymphocyte count <250 cells/mm^3^ [4], [5]. Because of a shortage of CD4-count measuring capacity in the country, most patients are started on ART based on clinical criteria only. Patients are seen at two weeks after ART initiation and then routinely every month for clinical assessment and ART-dispensing. Patients with drug side effects are changed to alternative ART regimens with zidovudine and/or efavirenz.

Malawi's national monitoring system for ART uses one patient master card for each patient and one ART register per facility [2], [3]. At enrolment, patient demographics, occupation, stage defining conditions and clinical stage are recorded on the master card and copied into the register. At every ART visit, follow-up details with their dates are entered in the master card, and these include transfer to another ART clinic, treatment discontinuation and death. Patients who fail to return for 3 months are marked as ‘lost to follow-up’. Although active follow-up of patients who fail to return to clinic has not been made mandatory due to resource constraints, more than half of the facilities in the national programme are consistently attempting to trace these patients through community visits. A patient cohort analysis is conducted at all sites every quarter, with clinic staff systematically reviewing and updating the latest follow-up status of all patients. The HIV Unit of the Ministry of Health and its partners conduct quarterly supervision and monitoring visits to all ART sites in the country. The supervisors check the accuracy, completeness and consistency of the register and master cards. Cohort analyses are checked and collected for aggregation and national level reporting.

A similar monitoring and supervisory system is used in the private sector, with the same monitoring tools and same quarterly supervision conducted by a clinical officer employed by the Malawi Business Coalition against AIDS. The only difference being that patients have to pay the equivalent of USD$3 per month for their ART, and this payment is similar regardless of the type of regimen.

### Data Collection and Analysis

The survey was conducted during supervision visits to all 103 public sector ART clinics and 38 private sector clinics, which took place between January and March 2007. In addition to the routine collection of data, all ART clinic registers and master cards were screened for army personnel who had accessed ART up to December 31^st^ 2006. For all army personnel identified, the following data were transcribed onto a structured form: site-specific registration number; sex; date, age and WHO clinical stage at ART initiation; date and type of follow-up outcome. The data were checked, entered and cleaned in MS Access and analysed using STATA 9.2. Observations were censored if patients were alive and on ART by 31^st^ December 2006. Adverse outcomes of death, lost to follow-up and ART discontinuation were regarded as ‘failure events’ and their dates were recorded, while transfers to other ART clinics were regarded as censoring events and their dates were also recorded. Categorical variables between army personnel and non-army personnel were analysed and compared using the chi squared test with odds ratios (OR). The probability of survival on ART was estimated using the Kaplan-Meier method. The level of significance for all comparisons was set at P = 0.05 or less, and 95% confidence intervals (CI) were used throughout.

Details were obtained at the 4 army ART clinics and all the other non-army ART clinics on the number of days in a week the clinic operated, the number of clinicians and nurses operating the clinic, drug stocks, and, in the last quarter of the year, drug adherence of patients alive and on ART. Drug adherence is assessed by ART staff every quarter by doing pill counts, with good drug adherence being equal to 95% or more of pills taken against the number that should have been consumed [5].

To measure the impact of ART on survival in the Malawi Defence Force, data on deaths amongst all army personnel in the country were obtained from the annual register of deaths in army personnel, which is maintained at the main army barracks in Lilongwe. The number of deaths each year for the last 5 years was documented.

### Ethical approval

General measures are provided in all ART facilities to ensure patient confidentiality, consent for HIV testing, and counselling and support for those who receive a positive HIV test result. Data for this study collected from ART sites and army records did not include any personal identifiers. The Malawi National Health Science Research Committee provides general oversight and approval for the collection and use of routine programmatic data for monitoring and evaluation, as was the case with this study. Formal ethical approval was therefore not required.

## Results

### General national patient cohort

By December 31^st^ 2006, a total of 81,821 patients had accessed ART at 103 public sector ART facilities in Malawi. Of these, 50,162 (61.3%) were female and 76,058 (93.0%) were aged 15 years or above. There were 9,833 (12%) patients started on ART in WHO clinical stage 1 or 2 with a CD4 count ≤250/mm^3^, and 71,988 (88%) started in stage 3 or 4. A total of 3,347 patients had accessed ART at 38 private sector ART facilities by the end of 2006. Of these, 1,635 (48.8%) were female and 3,201 (95.6%) were aged 15 years or above. There were 1,143 (34%) patients started on ART in WHO clinical stage 1 or 2 with a CD4 count ≤250/mm^3^, and 2,204 (66%) started in stage 3 or 4. Treatment outcomes by end of December 2006 are shown in **Table 1**. The routine quarterly cohort treatment outcomes in the public sector showed that 74% of 10,608 patients and 61% of 7,871 patients were alive and on ART at 6 and 12 months after enrolment respectively.

**Table 1:** National treatment outcomes of patients enrolled to antiretroviral therapy (ART) in the public sector, private sector and both sectors combined together, with outcomes censored on December 31^st^ 2006

	Public Sector	Private Sector	Both sectors
Patients enrolled	81,821	3,347	85,168
Alive and on therapy	57,356 (70%)	2,624 (78.4%)	59,980 (70.4%)
Dead	9,327 (11.4%)	222 (6.6%)	9,549 (11.2%)
Lost to follow-up (not seen at the clinic for 3 months)	7,753 (9.5%)	132 (3.9%)	7,885 (9.3%)
Stopped therapy	365 (0.5%)	7 (0.3%)	372 (0.4%)
Transferred out to another ART site	7,020 (8.6%)	362 (10.8%)	7,382 (8.7%)

### Army Personnel

Information on occupation was available for 82,297 (96.6%) patients in the national public and private sector cohort. There were 547 army personnel (0.7%), 526 men and 21 women. The mean age at ART initiation was 39 (range 24–65) years. Army personnel had accessed ART from 37 facilities; 543 army personnel had accessed ART from 34 sites in the public sector and 4 army personnel had accessed ART from 3 sites in the private sector; 406 (74%) of the patients were managed at the four army clinics.

Demographic and clinical details of army personnel and non-army personnel (the latter from the public sector) are shown in **Table 2**. There were significantly more males started on ART in the army compared with the general population. Significantly more army personnel accessed ART due to being in WHO clinical stage 1 or 2 with a CD4 count ≤250/mm^3^ compared with the general population accessing ART, but in those started on ART due to clinical illness there were more army personnel in WHO clinical stage 4.

**Table 2:** Demographic, clinical features and treatment outcomes censored on December 31^st^ 2006 in army personnel and non-army personnel starting free ART in the public sector in Malawi

	Army personnel	Non-army personnel	Odds ratio (95% CI)*
Number	547	81 274	
Males	526 (96.2%)	31 133 (38.3%)	40.3 [25.6–64.1]
Reasons for starting ART:			
WHO stage 1 and 2	119 (21.8%)	9 714 (12.0%)	2.1 [1.7–2.5]
WHO stage 3	248 (45.3%)	52 782 (64.9%)	0.45 [0.4–0.5]
WHO stage 4	180 (32.9%)	18 778 (23.1%)	1.6 [ 1.4–2.0]
Outcomes by December 2006			
Alive on ART at the clinic	365 (66.7%)	56 991 (70.1%)	
Transferred out to another clinic	98 (17.9%)	6 922 (8.5%)	2.3 [ 1.9–2.9]
Dead	71 (13.0%)	9 256 (11.4%)	
Lost to follow-up	9 (1.6%)	7 744 (9.5%)	0.2 [0.1–0.3]
Stopped therapy	4 (0.2%)	361 (0.5%)	

* Odds ratios shown for significant results only (p<0.05)

**Figure 1:**
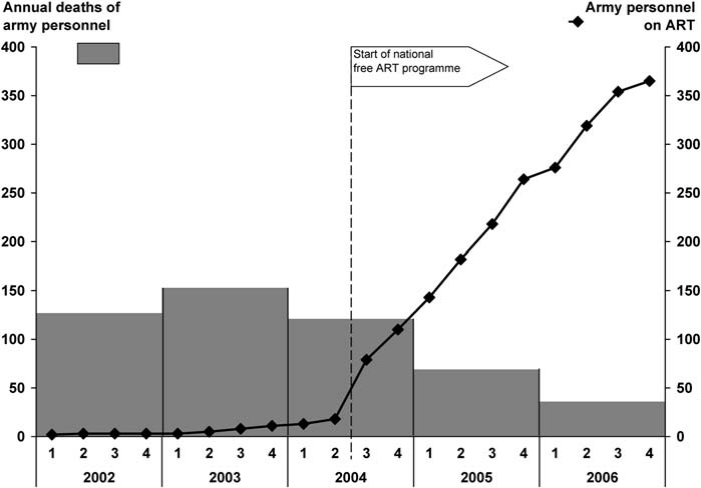
Number of army personnel alive and on antiretroviral therapy and the annual number of deaths of army personnel: 2002–2006.

The median observation time for the whole army cohort was 11.1 months (range 0.1 to 71 months), and the entire cohort had accumulated 596 person-years of observation. Treatment outcomes in army personnel by the end of December 2006 in army personnel are shown in **Table 2** **,** and are again compared with outcomes in non-army personnel in the public sector. There was a higher transfer out rate and a lower loss to follow-up rate amongst army personnel compared with the general population, but retention on therapy, deaths and stoppages of treatment were similar between the two groups. The number of army personnel retained alive and on ART by calendar period and the number of army personnel who died each year during the last 5 years is shown in **Figure 1** **.** The probability of being alive and on ART at 6, 12 and 18 months was 89.7%, 83.5%, and 78.5% respectively.

The 4 army ART clinics managed patients on an average of 3.0 days per week, similar to that seen in the 99 non-army clinics in the public sector (3.0 days per week). A total of 5 clinicians (1.25 per clinic) and 6 nurses (1.5 per clinic) provided services in the army clinics on the days they were open: this was similar to the 99 non-army clinics where a total of 150 clinicians (1.5 per clinic) and 155 nurses (1.5 per clinic) provided services. Of all patients alive and on ART in the army clinics in whom pill counts were done (n = 922, which includes army personnel and their relatives), 896 (97.2%) showed good drug adherence: this was significantly higher than that observed in patients alive and on ART in non-army clinics where 34,771 (92.7%) of 37,504 showed good drug adherence [OR 2.7, 95% CI 1.8–4.1, p<0.001]. Since national ART scale up started in Malawi, there have been no stock-outs of ART drugs in any facility, including the army clinics.

During 2002, before the start of the ART programme, there were 153 deaths among army personnel. In 2006, while staff numbers had remained similar, annual deaths had dropped to 36 (**Figure 1** **)**. This represents a dramatic 76% reduction in all-cause mortality between 2002 and 2006.

## Discussion

We believe that this is the first national survey in Sub-Saharan Africa of army personnel on ART examining uptake, retention on therapy and the impact that this has had over the last 5 years in reducing death rates. By the end of 2006, a total of 547 army personnel had been started on ART, the majority from the 4 army clinics that provide ART in Malawi. These army clinics operate on average the same number of clinic days per week and have the same ART clinic staffing complement as the other clinics around the country, and no clinic has experienced an ART drug stock out since national scale up started in 2004.

Army personnel differed from the general population accessing ART from public sector sites on several counts: there were more males; more patients started on ART due to being in stage 1 and 2 with a low CD4 count and due to being in WHO clinical stage 4; there was a low default rate and a higher transfer-out rate; and survival probabilities, at least up to 12–18 months were higher than for the general population. The findings in fact are more similar to those seen in the private sector in Malawi, and are not surprising. The majority of army personnel in Malawi are male. Because the main army clinics are situated in cities, there is relatively good access to laboratories that can perform CD4-lymphocyte tests and this may be one of the main reasons why a higher proportion of army personnel start ART due to being in WHO clinical stage 1 and 2. Why more army personnel start ART due to being in WHO clinical stage 4 is not known, but this may reflect a more comprehensive clinical assessment of patients. The Defence Force can maintain good follow-up of its patients, hence the low default rate. There was a high transfer rate due to frequent postings to different parts of the country. Recent operation research in Malawi (Yu, unpublished observations) shows that the majority of patients who transfer-out do in fact transfer-in at another site, and most of these patients subsequently stay alive and on therapy. Survival probabilities are good, probably due to larger numbers of the Defence Force accessing ART at an earlier stage of HIV disease and possibly due to the higher measured drug adherence in patients attending army clinics compared with that seen in the general population.

Without ART, HIV-infected patients assessed in WHO clinical stage 3 or 4 have a very poor prognosis in Malawi [6], while on ART the prognosis is transformed. Without the national ART program, it is likely that annual death rates in the army would have continued to increase over the last 5 years. With the ever increasing number of army personnel retained alive on treatment, this trend has been impressively reversed. This is corroborated by the 76% reduction in all-cause mortality between 2002 and 2006, which was observed independently in army staff records. It is likely that other occupational groups have benefited from a similar impact [7].

This operational study was based on data from the routine national monitoring system, and therefore has some limitations. Occupation was unknown for about 3% of patients, and this may have lead to a small undercounting of the number of army personnel who accessed ART in the country. It is also possible that some patients felt uncomfortable registering as a member of the Malawi Defence Force in ART clinics outside of the army health services, and may have given a different occupation to ART clinic staff. The average observation time of the national cohort of army personnel is still relatively short, and longer term outcomes could not be reliably assessed. The strengths of the study are that this is a full national survey, all ART-providing facilities in the public and private sector of Malawi were included, and the analysis is likely to provide near-complete information on all army personnel who have accessed treatment in the country, provided that they have been accurately recorded in the occupation column of the register. Malawi has established a standard, national monitoring system for ART used by all public and private sector facilities, the data are routinely validated during quarterly supervision through cross-checking of master cards and ART registers, ensuring high data quality. Occupation was available for 97% of patients and assuming that recording was accurate, the survey is likely to provide near-complete information on all army personnel started on ART in Malawi.

The Malawi Defence Force has shown outstanding leadership in its management of HIV/AIDS. First, it provided at its own expense ART for its personnel before the national scale up commenced. Second, it was pro-active in its desire to be part of the national ART scale up, with army medical personnel participating in the development and implementation of national plans and the national training curriculum. The four army clinics provide therapy to the majority of army personnel, and through a disciplined approach to follow-up the army has achieved excellent treatment outcomes. This approach should serve as an example to other defence forces in the world, as well as large companies in both the private and public sector.
